# Validation of a commercial antibody to detect endogenous human nicastrin by immunoblot

**DOI:** 10.12688/f1000research.19803.2

**Published:** 2020-01-10

**Authors:** Rosana A. Mesa, Elisha D.O. Roberson

**Affiliations:** 1 Department of Medicine, Washington University, St. Louis, MO, 63110, USA; 2 Department of Genetics, Washington University, St. Louis, MO, 63110, USA

**Keywords:** nicastrin, gamma secretase, polyclonal, human, western blot

## Abstract

Nicastrin (NCSTN) is a transmembrane glycoprotein that is part of the gamma-secretase complex. Gamma-secretase is a protease complex that cleaves type-I single-pass transmembrane proteins. There are many potential substrates for this complex, including NOTCH receptors and amyloid precursor proteins (APP). There are a number of commercial antibodies to nicastrin, but they do not agree on expected peptide size. We confirmed the specificity of a C-terminal binding rabbit anti-human antibody from Sigma-Aldrich (#N1660) using wildtype HEK293 cells and HEK293 cells deleted for nicastrin. The wildtype cells showed a prominent band at approximately 110 kDa. We confirmed this larger than expected sized was due to glycosylation by treating the lysate with peptide-N-glycosidase F (PNGase F), which reduced the band to less than 75 kDa. These data suggest that this polyclonal is specific for nicastrin and can detect endogenous levels of protein.

## Introduction

The γ-secretase complex is a multi-subunit, intramembrane protease (reviewed ^1^). It cleaves type-I single-pass transmembrane proteins within their transmembrane domain. This can lead to the release of an intracellular and an extracellular domain that may perform other functions. Examples include the cleavage of amyloid precursor protein (APP) to produce amyloid beta and the cleavage of activated NOTCH receptors to release their intracellular domain for translocation to the nucleus ^2^.

 Gamma-secretase is composed of several proteins, including a presenilin protease (PSEN1 or PSEN2), the presenilin enhancer gamma-secretase subunit (PEN2), an anterior pharynx-defective 1 protein (APH1A or APH1B), and nicastrin (NCSTN) ^3^. Nicastrin acquires extensive N-linked glycosylation during its maturation ^4, 5^, though the glycosylation may not be required for typical cleavage activity ^6^. The three-dimensional structure of human gamma-secretase shows that the heavily glycosylated ectodomain of nicastrin forms a horseshoe-like clamp on the extracellular portion of the complex ^7, 8^. It is thought that NCSTN may help control substrate selectivity ^9^. Understanding the role of nicastrin in gamma-secretase has been challenging. Gamma-secretase can cleave many substrates without nicastrin, though nicastrin does help to exclude some substrates via steric hindrance ^10– 12^. There are multiple commercial antibodies for NCSTN available, but they do not agree on the expected product size. We validated one commercial polyclonal antibody (#N1660; Sigma-Aldrich) using HEK293 wildtype and nicastrin knockout cells.

## Methods

### Antibody details

We used a commercially available rabbit anti-human IgG polyclonal antibody that targets human nicastrin (#N1660; Sigma-Aldrich, St. Louis, MO, USA; RRID: AB_477259) which is has performed well in some previous publications ^13, 14^. The antibody was raised against Uniprot nicastrin peptide Q92542 (709 amino acid total size). The polyclonal was generated by challenging rabbits with a synthetic peptide corresponding to the C-terminal cytoplasmic domain of nicastrin (peptides 693-709) fused with keyhole limpet hemocyanin as an adjuvant.

**Figure 1.**
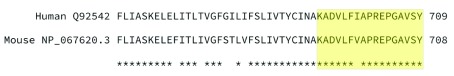
Human / mouse nicastrin alignment. Shown is a partial alignment between human and mouse nicastrin. The highlighted area represents the peptides use for generation of the polyclonal antibody. Asterisks represent a matching amino acid between the two sequences, and spaces are mismatches.

The technical documentation claims this subsequence is identical to the matching region of nicastrin in mouse. However, aligning Q92542 to the primary mouse nicastrin peptide sequence ( NP_067620.3) with Clustal Omega ^15, 16^ actually shows 1 mismatch (94.1% identity; Figure 1). It’s unclear if this discrepancy is due to changes to either the human or mouse peptide sequence for the most common isoform over time as the references have been updated.

We used a mouse anti-human beta actin monoclonal antibody (#AB6276; Abcam, Cambridge, MA, USA; RRID: AB_2223210) as a loading control. The details of all primary and secondary antibodies are summarized in Table 1.

**Table 1.** Details of the primary and secondary antibodies.

Antibody (Ab)	Manufacturer	Catalog Number	RRID	Lot number	Ab species	Ab type
Anti-Nicastrin	Sigma	N1660	AB_477259	076M4843V	rabbit	polyclonal
Anti-beta Actin (AC-15)	abcam	ab6276	AB_2223210	GR181659-16	mouse	monoclonal
Goat Anti-Rabbit IgG (H&L)- HRPO	Leinco Technologies	R115	AB_2810875	0117L320	goat	polyclonal
Goat Anti-Mouse IgG (H&L)- HRPO	Jackson ImmunoResearch	115-035-003	AB_10015289	129457	goat	polyclonal

### Cell lines and culture

We purchased the Human Embryonic Kidney cell line ( **HEK293**) from the ATCC (CRL-1573). We cultured all cells at 37°C and 5% CO _2_. For culture media, we used Dulbecco’s Modified Eagle’s Media ( **DMEM**; Gibco, Thermo-Fisher Scientific, #11965-084) supplemented with 5% Fetal Bovine Serum ( **FBS**; Gibco, Thermo-Fisher Scientific, #26140-079), 1% HEPES (Corning, #25-060-CI), 100 U/mL penicillin / streptomycin (Gibco, Thermo-Fisher Scientific, #15140-122), and 2 mM glutamine (Corning, #25-005-CI).

### HEK293 NCSTN knockout line

We used a HEK293 *NCSTN* knockout line we had previously generated using CRISPR/Cas9 genome-editing ^17^. Briefly, we synthesized our single-guide RNA as an IDT gBlock and cloned it into the pCR-Blunt TOPO vector. We co-transfected the single-guide RNA vector along with humanized Cas9 (RRID: Addgene_43861) into HEK293 cells, plated to single colonies, and screened for deleted clones by sequencing (Sequence Read Archive project PRJNA268374) and RT-qPCR (Data available from figshare, see source data ^18^). Full methodology for RT-qPCR is provided in the supplementary material of Cao *et al.* ^17^

### Cell line protein extraction

Reagent details can be found in Table 2 and Table 3. We harvested cells at ≥90% confluence and pelleted them by centrifugation at 4°C and 400 ×g for 5 minutes. We washed the cell pellet three times in 10 mL of cold phosphate buffered saline ( **PBS**). We then added 300 µL of cold lysis buffer (50 mM Tris-HCl pH 8.0, 2 mM EDTA, 150 mM NaCl, 1.0% NP-40, and 1.5% protease inhibitor cocktail) and lysed the cells with constant agitation for 30 minutes at 4°C. We removed insoluble debris by centrifugation for 15 minutes at 4°C and 10,400 ×g. We determined the concentration of the cleared lysates using a Pierce BCA assay kit (#23227). We stored the lysates in aliquots at -80°C until further use.

### Mouse liver protein extraction

We received a snap-frozen mouse liver (2 month-old C57BL/6 mouse) from the Alfred Kim lab, which had been obtained according to their approved IACUC protocol. We minced the liver into pieces and homogenized in ice-cold lysis buffer (5 mM Tris-HCl pH 8.0, 250 mM sucrose, 5 mM EDTA, 1.5% protease inhibitor cocktail) using a Wheaton tissue grinder. We then passed the solution through a QIAshredder spin-column (Qiagen #79656) to facilitate more complete lysis. We spun cellular debris out of solution by a 5 minute spin at 5,000 xg. We then precipitated membrane-enriched fragments by spinning for 5 minutes at 11,000 xg (4°C), then spinning the supernatant for an additional 1 hour at 4°C and 11,000 xg. We then extracted proteins from the membrane pellet by resuspending in buffer containing 2% (v/v) Triton X-100 and incubating on ice for 30 minutes. Any remaining unlysed material was pelleted with a 1 hour, 4°C, 11,000 xg spin. We determined the protein concentration of the lysate using the Pierce BCA assay kit. We stored lysates in aliquots at -80°C until blotting.

**Table 2.** Details of Cell lysis reagents.

Reagent	Manufacturer	Catalog Number
1M Tris–HCl pH 8	Corning	46-031-CM
0.5M EDTA pH8	Corning	46-034-Cl
5M NaCl	Sigma	S5150
Surfact-Amps NP-40 Detergent Solution	Thermo Scientific	85124
Protease Inhibitor Cocktail	Sigma	P8340
Sucrose	Sigma	S1888
Triton X-100	Sigma	T8787

**Table 3.** Details of SDS-PAGE / Immunoblotting reagents.

Protocol Steps	Reagents	Manufacturer	Catalog Number
Protein concentration measurement	Pierce BCA Protein assay kit	Thermo Scientific	23227
Cell lysate preparation	2x Laemmli sample buffer β-mercaptoethanol	Biorad Sigma	161-0737 M3148
Electrophoresis	7.5% Mini-PROTEAN TGX gel, 10wl, 30 µl 10X Tris/Glycine/SDS Buffer	Biorad Biorad	4561023 161-0732
Immunoblotting	Immobilon-P PVDF Membrane (0.45 µm) 2-Propanol 10X Tris/Glycine Buffer 10X TBST	Millipore Sigma Biorad EZ BioResearch	IPVH08100 190764 161-0734 S-1012
Chemiluminescence reaction	SuperSignal West Pico Chemiluminescent Substrate	Thermo Scientific	34080

### Enzymatic deglycosylation

We used peptide-N-glycosidase F ( **PNGase F**; #P0704S; New England Biolabs, Ipswich, MA, USA) to remove N-linked sugars. We denatured about 50 µg of protein in glycoprotein denaturing buffer (included with NEB kit; 0.5% SDS, 40 mM DTT) at 100°C for 10 minutes, and then incubated the lysate with PNGase F for 3 hours at 37°C, according to the manufacturer’s instructions. We treated a control in parallel under the same conditions, but omitted the PNGase F enzyme.

### Immunoblotting

We denatured the protein lysate by boiling for 5 minutes in Laemmli sample buffer (5% β-mercaptoethanol). We resolved the proteins on precast 7.5% polyacrylamide gels (Mini-protean TGX; Bio-Rad, Hercules, CA, USA) after loading approximately 20 µg of lysate. We used the Precision Plus Dual-Color Standard as a molecular weight marker (Bio-Rad, Hercules, CA). We prepared PVDF membranes (0.45 µm) by incubating 2 minutes in 100% isopropanol, washing in Milli-Q water for 2 minutes, and equilibrating in transfer buffer for 10 minutes. We transferred separated proteins to the PVDF membrane in transfer buffer without methanol at 200 mA for 2 hours. We blocked the membrane by incubating in blocking buffer (TBST with 5% skim milk powder) for 1 hour at room temperature with gentle rocking. We probed the membrane using primary antibodies to nicastrin (1/1000) and beta actin (1/5000) diluted in blocking buffer overnight at 4°C with gentle rocking. We removed excess unbound antibody by rinsing the membranes 5 times for 10 minutes each in TBST buffer. The anti-mouse and anti-rabbit secondary antibodies were both conjugated to horseradish peroxidase ( **HRP**). We incubated the membranes with secondary antibody (1/7000) in blocking buffer for 1.5 hours at room temperature, followed by washing 5 times for 10 minutes each in TBST. We used the Supersignal West Pico Chemiluminescent Substrate reagent (ThermoFisher, Waltham, MA) to detect secondary antibodies.

## Results

### The nicastrin polyclonal binds to endogenous nicastrin in HEK293 extracts

**Figure 2.**
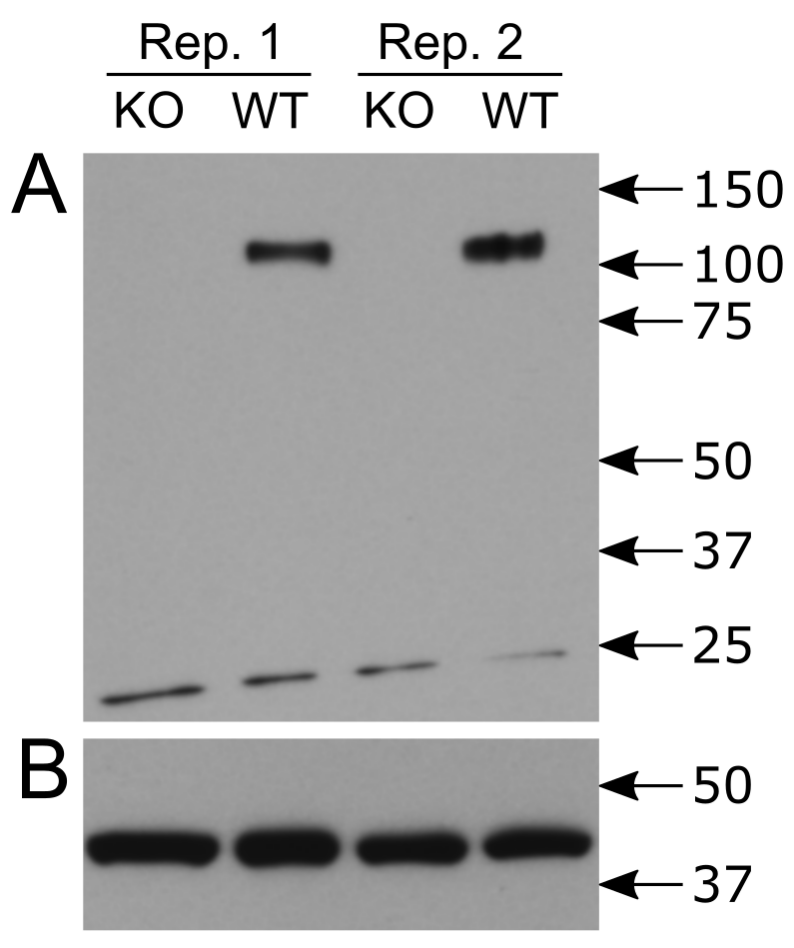
Immunoblot of endogenous nicastrin. **A**. The NCSTN antibody binds to endogenous levels of protein in wildtype (WT) HEK293 cells with a band at ~110 kDa. The band is absent in NCSTN knockout (KO) cells. Both replicates show an unidentified band at 25 kDa. **B**. The actin antibody shows the expected ~42 kDa band in both replicates of wildtype and knockout cells. Abbreviations: rep., replicate.

We collected protein lysates from wildtype HEK293 cells and HEK293 *NCSTN* knockouts. The manufacturer provided example blots were derived from HEK293 cells, but used an overexpression construct. In wildtype HEK293 cell lysates, a single, strong band at ~110 kDa can be seen on the blot, and this band is missing in the nicastrin knockout line lysates ( Figure 2A, underlying data ^19, 20^). The loading controls for the wildtype replicates and knockout replicates all show the expected band for actin ( Figure 2B, underlying data ^19, 20^), supporting that the loss of the nicastrin band is specific to the knockout and not a loading error. It is worth noting that despite a low background, the nicastrin blots showed an approximately 25 kDa band in both wildtype and knockout lysates. We searched the protein sequence used to develop the antibody (KADVLFIAPREPGAVSY) with protein blast using the *Homo sapiens* non-redundant peptide database automatically adjusted for short queries, but only matches to nicastrin had a reasonable e-value (2×10 ^-9^ to 7×10 ^-11^). It is therefore unclear if this band is from a non-specific contaminant in the antibody, a similar peptide that is poorly annotated in the non-redundant protein database, or a nicastrin degradation product.

### The larger than expected band size for nicastrin is due to glycosylation

The nicastrin antibody documentation lists the expected fragment size as approximately 110 kDa, and this band size was confirmed on our blots. However, calculating the fragment size of human nicastrin protein sequence Q92542 using Expasy tools ^21^ gives an estimated 78.4 kDa size for the nascent fragment and a reduced 75.2 kDa size after cleavage of the signal peptide. We hypothesized this discrepancy might be due to glycosylation.

**Figure 3.**
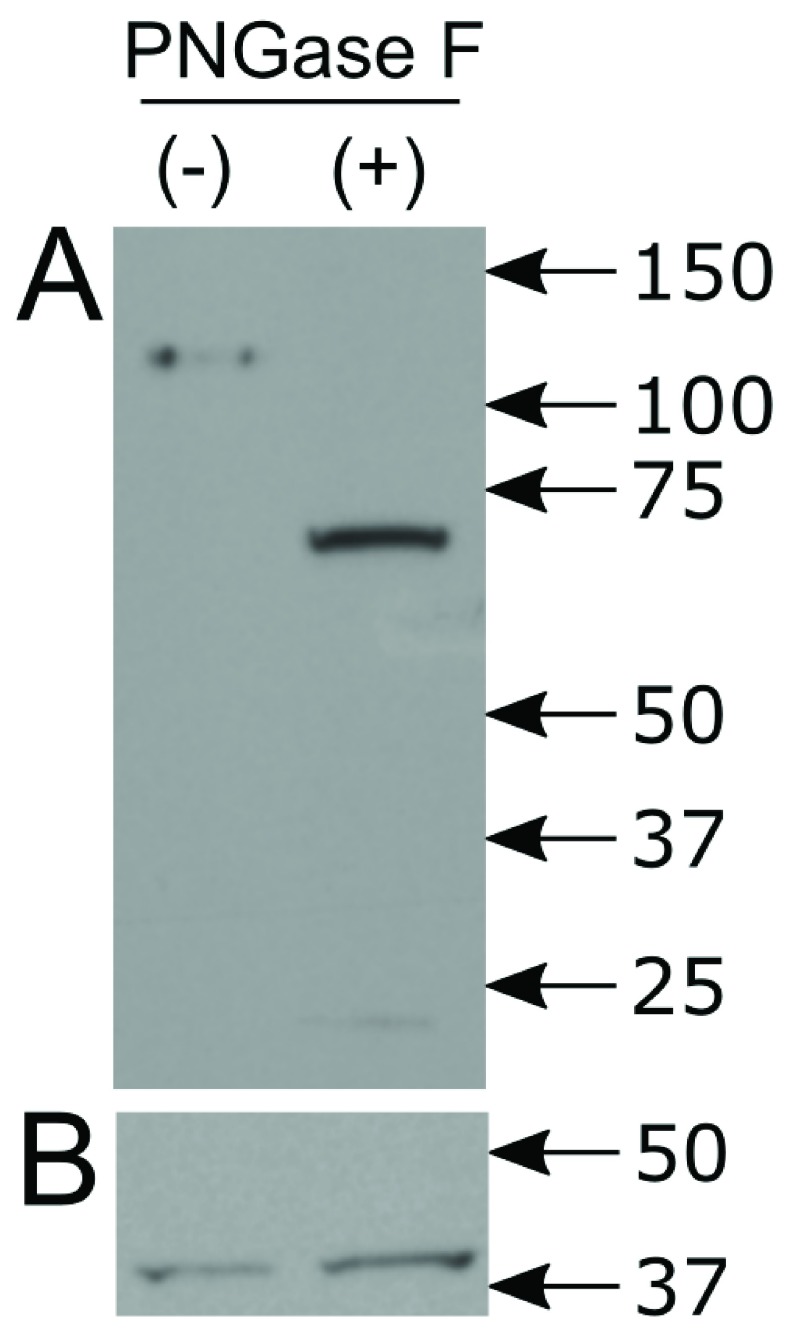
Nicastrin immunoblot with PNGase F treatment. **A**. In lysates untreated with PNGase F (-), the expected ~110 kDa band is present. With PNGase F treatment (+), the band regresses to less than 75 kDa. **B**. In both PNGase treated and untreated lysates, the beta actin band is unchanged.

 We tested this hypothesis by first treating the lysates PNGase F, which will release asparagine-linked oligosaccharides. This reduced the molecular weight of the nicastrin band to less than 75 kDa ( Figure 3A, underlying data ^22, 23^) without affecting the actin band ( Figure 3B underlying data ^22, 23^). This phenomenon of a smaller than expected nicastrin band has been observed previously ^6, 24^. It is possible that a longer signal sequence than expected is cleaved from the nascent peptide. Given that detailed information is available for the signal cleavage of nicastrin ^9^, a more likely explanation might be that the charge profile of the polypeptide affects its migration.

### polypeptide affects its migrationThe antibody binds to endogenous mouse nicastrin

**Figure 4.**
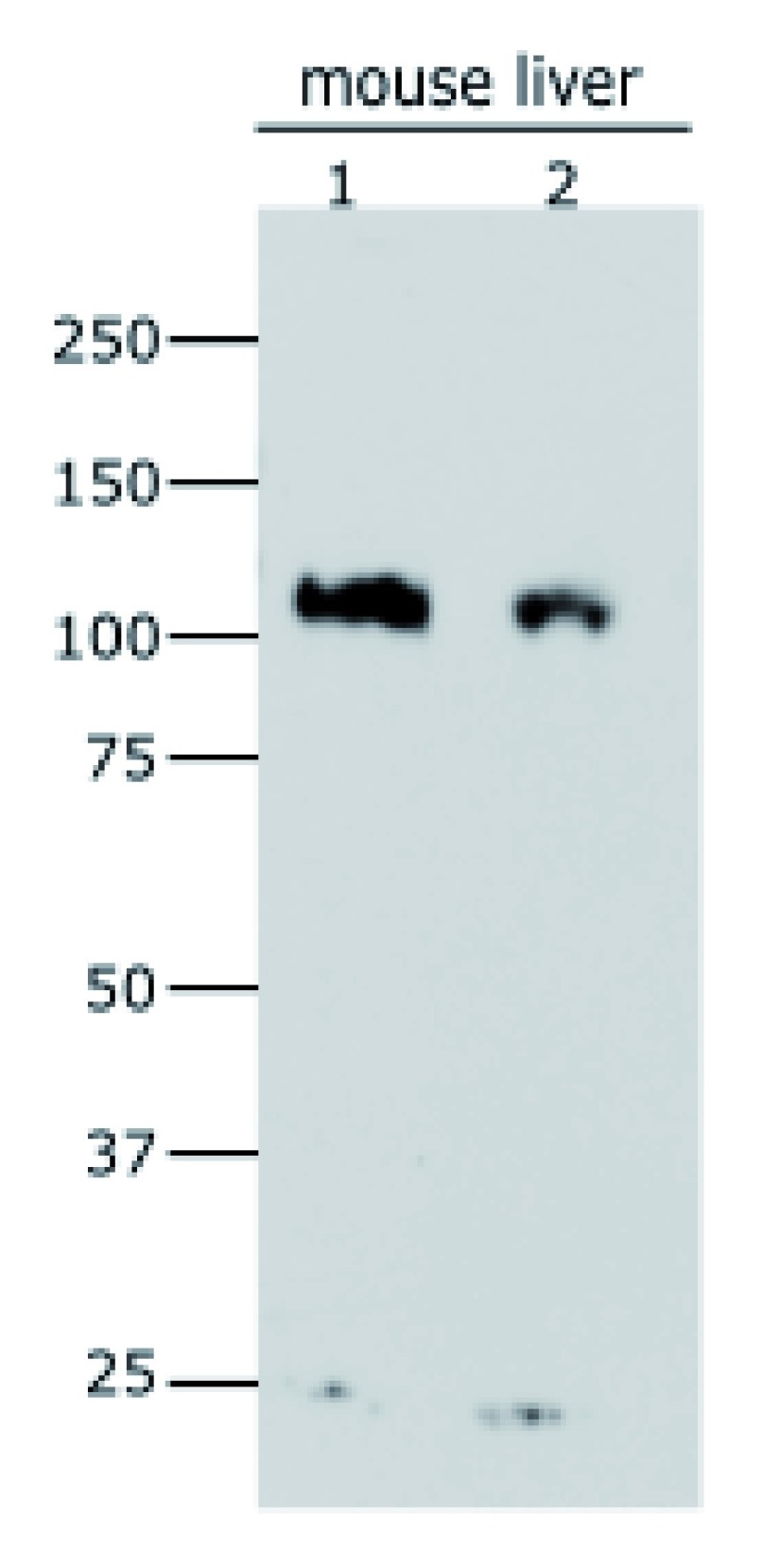
Immunoblot of murine nicastrin. Blot showing the results for 35 µg (1) or 25 µg (2) of mouse membrane protein lysate. The expected ~110 kDa band for mature nicastrin is present, as is the non-specific band present in most blots at < 25 kDa. These data suggest the antibody works as well for murine nicastrin as it does for human nicastrin.

As noted above, there were mismatches between the sequence used to generate the antibody and the mouse sequence for nicastrin. It was possible that this mismatch was enough to reduce the effectiveness of this antibody in mouse extracts. We extracted protein from frozen mouse liver to test this possibility. We were able to confirm the presence of a band of the expected size in the mouse extracts ( Figure 4, underlying data ^25^). The same small, non-specific band was present in these blots as well.

## Conclusion

We tested by immunoblot an anti-nicastrin antibody using HEK293 cell lysates and mouse liver extracts. Our results show that the antibody is sensitive enough to detect endogenous protein with reasonable specificity. It is able to bind to both glycosylated nicastrin and nicastrin without sugar linkages. The antibody functions for both endogenous human and mouse protein. It is unclear how well the antibody would work for cell staining due to the non-specific 25 kDa band we observed on nicastrin blots. Based on these data obtained with the protocols described above, we can confirm the utility of this nicastrin antibody for immunoblotting.

## Data availability

### Source data

Home sapiens HEK293 NCSTN knockout by Cas9, Accession number: PRJNA268374

Figshare: HEK293 nicastrin knockout RT-qPCR. https://doi.org/10.6084/m9.figshare.7578539.v1 ^18^

This project contains the following source data:

- knockout_rtqpcr.csv (Raw Ct values of RT-qPCR confirming the knockout (CRISPR-Cas9 mediated) of nicastrin in HEK293 cells.)

Data are available under the terms of the Creative Commons Attribution 4.0 International license (CC-BY 4.0).

### Underlying data

Figshare: NCSTN antibody validation - actin antibody in HEK293 knockout line. https://doi.org/10.6084/m9.figshare.8952968.v1 ^19^

This project contains the following underlying data:

- AntibodyValidation_NCSTN_KO_actin_Ab.svg (TIF image of actin antibody blot stored in a scaleable vector graphic file)

Figshare: NCSTN antibody validation - NCSTN antibody in HEK293 knockout line. https://doi.org/10.6084/m9.figshare.8952953.v1 ^20^

This project contains the following underlying data:

- AntibodyValidation_NCSTN_KO_NCSTN_Ab.svg (TIF image of NCSTN antibody blot stored in a scaleable vector graphic file)

Figshare: NCSTN antibody validation - actin antibody in HEK293 knockout line after PNGase treatment. https://doi.org/10.6084/m9.figshare.8952983.v1 ^22^

This project contains the following underlying data:

- AntibodyValidation_NCSTN_PNGase_actin_Ab.svg (TIF image of actin antibody blot stored in a scaleable vector graphic file)

Figshare: NCSTN antibody validation - NCSTN antibody in HEK293 knockout line after PNGase treatment. https://doi.org/10.6084/m9.figshare.8952977.v1 ^23^

This project contains the following underlying data:

- AntibodyValidation_NCSTN_PNGase_NCSTN_Ab.svg (TIF image of NCSTN antibody blot stored in a scaleable vector graphic file)

Figshare: NCSTN antibody validation - NCSTN antibody with murine liver protein. http://www.doi.org/10.6084/m9.figshare.11541864.v1 ^25^

This project contains the following underlying data:

- SuppFig5_MouseNCSTN.svg (TIF image of NCSTN antibody blot stored in a scaleable vector graphic file)

Data are available under the terms of the Creative Commons Zero "No rights reserved" data waiver (CC0 1.0 Public domain dedication).
