# Supplementary material for: Validation of a commercial antibody to detect endogenous human nicastrin by immunoblot
Source: F1000Res. 2020 Jan 10;8:1211. Originally published 2019 Jul 29. [Version 2] doi: 10.12688/f1000research.19803.2 (PMC7194342; doi:10.12688/f1000research.19803.2)
Supplement: Peer review history

# Peer review: Reviewer response for version 2

**DOI:** 10.5256/f1000research.24258.r61941

**Published:** 2020-04-15

**Authors:** Jongkyun Kang

**Affiliations:** 1 Department of Neurology, Brigham and Women’s Hospital, Harvard Medical School, Boston, MA, USA

This article validated several commercial antibodies against Nicastrin protein which is one of the gamma-secretase complex. Authors used different cell types and organisms to validate antibodies for the specificity and sensitivity. 

 As other reviewers pointed out, this article has less interesting since other papers already showed the specificity of several antibodies in human or mouse cell line or tissues. Furthermore, postmodification mechanism of Nicastrin by glycosylation was also well established. 

 Despite less novelty, this article would be helpful for the community who have a plan to perform a Western analysis on HEK293 cell line which is major experimental condition of this paper.

Is the work clearly and accurately presented and does it cite the current literature?

Partly

Are all the source data underlying the results available to ensure full reproducibility?

Yes

Are sufficient details of materials, methods and analysis provided to allow replication by others?

Yes

Are the conclusions drawn adequately supported by the results?

Yes

Reviewer Expertise:

Molecular biology, Biochemistry, Neuropathology

I confirm that I have read this submission and believe that I have an appropriate level of expertise to confirm that it is of an acceptable scientific standard.

# Peer review: Reviewer response for version 2

**DOI:** 10.5256/f1000research.24258.r58489

**Published:** 2020-01-31

**Authors:** Satoru Funamoto

**Affiliations:** 1 Laboratory of Neuropathology, Graduate School of Life and Medical Sciences, Doshisha University, Kyoto, Japan

This reviewer thinks that this manuscript is sufficient for the F1000Research.

Is the work clearly and accurately presented and does it cite the current literature?

No

Are all the source data underlying the results available to ensure full reproducibility?

Yes

Are sufficient details of materials, methods and analysis provided to allow replication by others?

Yes

Are the conclusions drawn adequately supported by the results?

Yes

Reviewer Expertise:

Neuropathology and biochemistry

I confirm that I have read this submission and believe that I have an appropriate level of expertise to confirm that it is of an acceptable scientific standard.

# Peer review: Reviewer response for version 1

**DOI:** 10.5256/f1000research.21724.r51771

**Published:** 2019-09-18

**Authors:** Lucia Chavez-Gutierrez

**Affiliations:** 1 VIB‐KU Leuven Center for Brain & Disease Research, Leuven, Belgium

The authors validated a commercially available rabbit polyclonal anti-Nicastrin antibody from Sigma-Aldrich (#N1660) in immunoblot analysis, using as inputs total lysates from wildtype and Nicastrin knockout HEK293 cell lines. The N1660 antibody, generated against a synthetic peptide corresponding to the C-terminal cytoplasmic part of human Nicastrin (693-709), identified a band at ~110 kDa only in the wild type lysate and another band at ~ 25 kDa in both wildtype and knockout lysates. The data does not clarify whether the low-molecular weight signal is a non-specific band or a nicastrin-derived peptide.

 Treatment with PNGnase F to remove all N-linked oligosaccharide chains, reduced the molecular weight of the nicastrin band to less than 75 kDa, without affecting the loading control actin band. The observed shift in molecular weight is supported by previous observations (Herreman A. *et al.*, 2003 ^1^) and the authors should acknowledge this study.

**Other Points:**

- The authors should test the N1660 antibody against mouse Nicastrin.
- “It is thought that NCSTN may help control substrate selectivity.” The ‘substrate receptor” model (Shah *et al*, 2005 ^2^) has been challenged by independent studies (Chávez‐Gutiérrez *et al*, 2008 ^3^; Zhao *et al*, 2010 ^4^), and it is not supported by available high resolution structural data for the gamma secretase complex (Bai *et al*, 2015 ^5^). More recent studies indicate that the ectodomain of NCT has both passive and active roles in gamma secretase-mediated proteolysis. On one hand, it restricts the access of substrates presenting large ectodomains by steric hindrance (Bolduc *et al*, 2016 ^6^). On the other, it has an active role in the regulation of the sequential proteolysis of APP and response to gamma secretase modulators (GSMs); thus, nicastrin modulates Aβ length. 
- “This phenomenon of a smaller than expected nicastrin band has been observed previously. It is possible that a longer signal sequence than expected is cleaved from the nascent peptide, or the charge profile of the polypeptide affects its migration.” Shah *et al.*, 2005 ^2^ provides information about the precise signal peptide sequence cleaved from the nascent NCT polypeptide.

Is the work clearly and accurately presented and does it cite the current literature?

Partly

Are all the source data underlying the results available to ensure full reproducibility?

Yes

Are sufficient details of materials, methods and analysis provided to allow replication by others?

Yes

Are the conclusions drawn adequately supported by the results?

Yes

Reviewer Expertise:

Biochemistry, Intramembrane proteolysis, Alzheimer's Disease

I confirm that I have read this submission and believe that I have an appropriate level of expertise to confirm that it is of an acceptable scientific standard, however I have significant reservations, as outlined above.

## References

[1] : gamma-Secretase activity requires the presenilin-dependent trafficking of nicastrin through the Golgi apparatus but not its complex glycosylation. *J Cell Sci*.2003;116(Pt 6) : 10.1242/jcs.00292 1127-36 10.1242/jcs.00292 12584255

[2] : Nicastrin functions as a gamma-secretase-substrate receptor. *Cell*.2005;122(3) : 10.1016/j.cell.2005.05.022 435-47 10.1016/j.cell.2005.05.022 16096062

[3] : Glu(332) in the Nicastrin ectodomain is essential for gamma-secretase complex maturation but not for its activity. *J Biol Chem*.2008;283(29) : 10.1074/jbc.M803040200 20096-105 10.1074/jbc.M803040200 18502756

[4] : Gamma-secretase composed of PS1/Pen2/Aph1a can cleave notch and amyloid precursor protein in the absence of nicastrin. *J Neurosci*.2010;30(5) : 10.1523/JNEUROSCI.3826-09.2010 1648-56 10.1523/JNEUROSCI.3826-09.2010 20130175PMC2853942

[5] : An atomic structure of human γ-secretase. *Nature*.2015;525(7568) : 10.1038/nature14892 212-217 10.1038/nature14892 26280335PMC4568306

[6] : Nicastrin functions to sterically hinder γ-secretase-substrate interactions driven by substrate transmembrane domain. *Proc Natl Acad Sci U S A*.2016;113(5) : 10.1073/pnas.1512952113 E509-18 10.1073/pnas.1512952113 26699478PMC4747693

# Response

**Published:** 2020-01-08

**Authors:** Elisha Roberson

**Affiliations:** Washington University in St. Louis, USA

Reviewer question 1: Treatment with PNGnase F to remove all N-linked oligosaccharide chains, reduced the molecular weight of the nicastrin band to less than 75 kDa, without affecting the loading control actin band. The observed shift in molecular weight is supported by previous observations (Herreman A. *et al.*, 2003) and the authors should acknowledge this study.

**Response: This is a valid point, and have cited the appropriate study. Thanks for the suggestion.**

 Reviewer question 2: The authors should test the N1660 antibody against mouse Nicastrin.

**Response: As requested we used murine protein to test the antibody. This data has been added as Figure 4, and the expected band size was observed, along with the non-specific small molecular weight band that show up in the HEK293 blots.**

 Reviewer question 3: "It is thought that NCSTN may help control substrate selectivity.” The ‘substrate receptor” model (Shah *et al*, 2005) has been challenged by independent studies (Chávez‐Gutiérrez *et al*, 2008; Zhao *et al*, 2010), and it is not supported by available high resolution structural data for the gamma secretase complex (Bai *et al*, 2015). More recent studies indicate that the ectodomain of NCT has both passive and active roles in gamma secretase-mediated proteolysis. On one hand, it restricts the access of substrates presenting large ectodomains by steric hindrance (Bolduc *et al*, 2016). On the other, it has an active role in the regulation of the sequential proteolysis of APP and response to gamma secretase modulators (GSMs); thus, nicastrin modulates Aβ length.

**Response: These are all excellent points. It is, of course, not possible to exhaustively discuss all the possibilities for nicastrin function in gamma-secretase in the introduction, as that is more suitable to a full literature review. However, these are points worth at least mentioning, and good references to support the paper. We have revised the paper to point to some of these different evidence sets regarding nicastrin function and have added the suggested references.**

 Reviewer question 4:  “This phenomenon of a smaller than expected nicastrin band has been observed previously. It is possible that a longer signal sequence than expected is cleaved from the nascent peptide, or the charge profile of the polypeptide affects its migration.” Shah *et al.*, 2005 provides information about the precise signal peptide sequence cleaved from the nascent NCT polypeptide.

**Response: Given the work from Shah determining the signal peptide sequence, we have revised this section to point out this information (referencing the paper). We have also reworded to suggest that perhaps the disparity is more likely to be due to conformation / charge profile than additional signal peptide sequence cleavage.**

# Peer review: Reviewer response for version 1

**DOI:** 10.5256/f1000research.21724.r51767

**Published:** 2019-08-06

**Authors:** Satoru Funamoto

**Affiliations:** 1 Laboratory of Neuropathology, Graduate School of Life and Medical Sciences, Doshisha University, Kyoto, Japan

It is very important to validate commercial antibodies. However this reviewer thinks it would help to enhance the value of this manuscript if authors consider the below:

1. Sigma’s anti-Nicastrin antibody has been already a golden standard for Nicastrin detection. It is not clear how other commercial antibodies do not agree on size. It is very important to show bands detected with several commercial anti-Nicastrin antibodies. This could be very informative.
2. It is already published that PNGase and Endo H treatments altered migration distance of Nicastrin in gels ^1^.

Is the work clearly and accurately presented and does it cite the current literature?

No

Are all the source data underlying the results available to ensure full reproducibility?

Yes

Are sufficient details of materials, methods and analysis provided to allow replication by others?

Yes

Are the conclusions drawn adequately supported by the results?

Yes

Reviewer Expertise:

Neuropathology and biochemistry

I confirm that I have read this submission and believe that I have an appropriate level of expertise to confirm that it is of an acceptable scientific standard, however I have significant reservations, as outlined above.

## References

[1] : gamma-Secretase activity requires the presenilin-dependent trafficking of nicastrin through the Golgi apparatus but not its complex glycosylation. *J Cell Sci*.2003;116(Pt 6) : 10.1242/jcs.00292 1127-36 10.1242/jcs.00292 12584255

# Response

**Published:** 2020-01-08

**Authors:** Elisha Roberson

**Affiliations:** Washington University in St. Louis, USA

Reviewer question 1: Sigma’s anti-Nicastrin antibody has been already a golden standard for Nicastrin detection. It is not clear how other commercial antibodies do not agree on size. It is very important to show bands detected with several commercial anti-Nicastrin antibodies. This could be very informative.

**Response: We agree that a comprehensive screening of multiple antibodies would be fruitful. For this experiment we focused on validating a commonly used antibody to ensure that it was suitable for our future experimental designs. We are considering this project in the future, given that we can generate the necessary funds to purchase the requisite antibodies.**

 Reviewer question 2: It is already published that PNGase and Endo H treatments altered migration distance of Nicastrin in gels.

**Response: It has been previously published in your suggested reference from 2003. We are able to confirm that this effect still persists, and that >15 years later the antibody specificity is still acceptable. We appreciate the suggestion for this reference and have appropriately cited it in the revised version.**
